# Recurrent venous thrombosis in an adequately anticoagulated patient with pemphigus vulgaris

**DOI:** 10.1186/s12959-016-0080-6

**Published:** 2016-03-04

**Authors:** Paul R. J. Ames, Maria Graf, Fabrizio Gentile

**Affiliations:** Immune Response and Vascular Disease Unit, Nova University, Lisbon, Portugal; Dipartimento di Medicina Molecolare e Biotecnologie Mediche, Universita Federico II, Napoli, Italy; Dipartimento di Medicina e Scienze della Salute, Universita’ del Molise, Campobasso, 86100 Italy

**Keywords:** Pemphigus vulgaris, Thrombosis

## Abstract

**Background:**

Several autoimmune skin disorders are characterised by an increased risk of thrombosis, with bollous pemphigoid carrying a higher risk than pemphigus vulgaris (PV). We describe the case of a middle aged gentleman who developed recurrent venous thromboembolism despite adequate oral anticoagulation during very active PV that required escalation of treatment to bring the disease under control.

**Case presentation:**

In May 2014 a 49 year gentleman was admitted for widespread mucocutaneous blistering diagnosed as PV by histology and immunofluorescence. After 6 weeks of treatment with systemic steroids and azathioprine the patient developed pulmonary emboli and started oral anticoagulation with warfarin. In late September, the patient re-presented with a severe flare of PV and a recurrent deep vein thrombosis despite oral anticoagulation within therapeutic range. Warfarin was changed to subcutaneous low molecular heparin in therapeutic dose while treatment for pemphigus was escalated: first azathioprine was switched to mycophenolate mofetil and the steroids dose increased; then due to poor response, intravenous immunoglobulins were given for three courses and finally he received four infusions of Rituximab that induced sustained remission. In April 2015 the dose of mycophenolate was decreased but anticoagulation was continued until the beginning of July 2015 to ensure that decreasing immune suppression did not allow the emergence of another flare with attendant thrombotic risk.

**Conclusion:**

The case highlights the risk of thrombosis and re-thrombosis in aggressive PV and demands further clinical research in this area to assess the need for thromboprophylaxis in aggressive bollous skin disease.

## Background

Pemphigus vulgaris (PV) is an autoimmune skin disorder characterized clinically by intra epidermal blisters and erosions due to an immunoglobulin G autoantibody with specificity against the desmosomal desmogleins 3 and 1 [1]. PV is also characterised by an increased thrombotic risk as highlighted by the Oxford Record Linkage Study in PV patients admitted to hospital [2], though coagulation activation in PV is not enhanced as in bullous pemphigoid [3]. We describe herein a patient with PV who developed pulmonary embolism (PE) first then recurrent venous thromboembolism (VTE) despite adequate oral anticoagulation during the course of very active PV.

## Case presentation

**Fig. 1:**
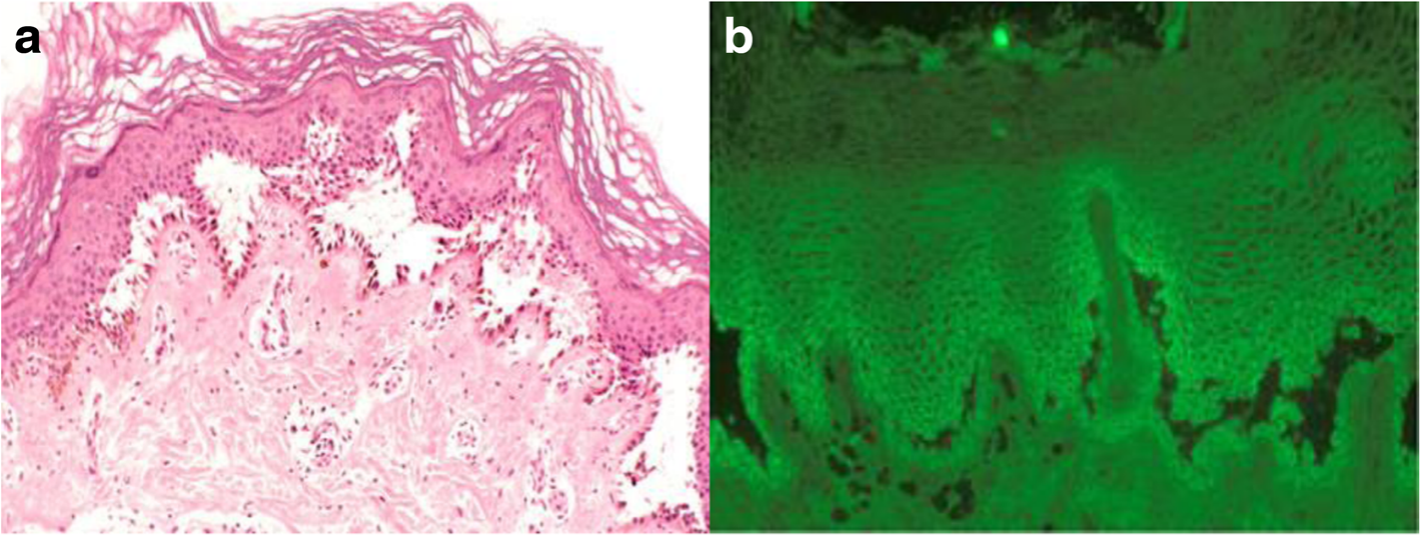
Histology of skin biopsy showing: **a**) Light microscopy showing intradermal acantolytic blisters; **b**) Immunofluorescence showing intradermal cellular staining for desmogleins 3

A 49 year gentleman was admitted in May 2014 to the dermatology ward for widespread blistering around his groins then spreading to mouth, nose, torso, penis and scalp. A skin biopsy revealed intradermal acantolytic blisters by conventional microscopy and strong intradermal cellular staining for pemphigus antigens by immunofluorescence microscopy in keeping with PV (Fig. 1). ANA, c-ANCA and p-ANCA were negative. He commenced treatment with topical and systemic steroids and azathioprine. Before this admission he had always been fit and well, with no personal or family history of VTE and was ambulant during his admission. Six weeks later the patient developed bilateral PE and started anticoagulation with therapeutic Deltaparin switched then to warfarin that was monitored in the community at fortnightly intervals with international normalised ratio (INR) between 2.3 and 2.9. After 9 weeks, in late September, the patient re-presented with a severe flare of PV and a swollen left leg despite being in an adequate therapeutic range, the INR being 2.5 two weeks before recurrent event and INR 2.7 at recurrent event: a doppler ultrasound revealed a superficial femoral vein occlusion. The dose of systemic steroids was increased and azathioprine was switched to mycophenolate mofetil 1.5 g bd: because his recurrent VTE happened whilst he was in therapeutic INR, warfarin was switched to low molecular weight heparin at treatment dose to be continued for no less than 6 months from the VTE. Owing to poor PV response in late October the patient received one monthly infusion of intravenous immunoglobulin (0.5 mg/kg over 5 days) for a total of three courses. In January 2015 he received four infusions of Rituximab (375 mg/m^2^) at weekly intervals that brought his PV under control. He remained on oral mycophenolate 1.5 g bd until April 2015 when it was decreased to 1.0 g bd: to ensure that the decrease of immunosuppressant dose did not favour a disease flare with a new recurrent VTE his anticoagulation was continued up to the beginning of July 2015 instead of stopping after 6 months. A thrombophilia screen including Factor V Leiden, Prothrombin 20210 mutation, antithrombin, protein C and S, anticardiolipin antibodies and lupus anticoagulant performed after cessation of anticoagulation was normal. At his last follow up in mid-December 2015 the patient was in sustained remission on mycophenolate 1.0 g bd.

## Conclusion

Our patient developed PE during the initial phase of his extensive and aggressive PV that was initially managed with azathioprine and steroids plus warfarin leading to a transient remission. At 9 weeks from the PE the patient represented with a severe flare of PV and a recurrent VTE despite adequate anticoagulation. The activity of PV required several sequential changes and/or addition of medications before it was decided to offer him Rituximab not without some concerns [4] but this finally brought the patient into remission. At the recurrence of VTE direct oral anticoagulants were not taken into account to minimise the perceived bleeding risk on a mucosa and skin that were still actively inflamed and to avoid the remote possibility of a skin reaction; hence the switch from warfarin to low molecular weight heparin. The negative thrombophilia screen strengthens the case of our active PV patient being in a pro-thrombotic state despite a study showing that neither active or inactive PV were associated with coagulation activation (3).

Other cases of VTE have been described in association with PV in the English literature: these affected lungs [5, 6], lung with deep veins, cardiac chambers with myocardial infarction [7] and a further VTE attributed to the use of intravenous immunoglobulins to treat PV [8]. The odds of developing VTE in PV are 3.28 according to a record-linkage study over the period 1999 to 2008 [2]. This is the first case of recurrent VTE developing in a patient with PV despite adequate anti vitamin K anticoagulation and with no other VTE risk factors. In the light of ours and other available cases and owing to the elevated odds of the record linkage study, further studies are needed to evaluate whether thromboprophylaxis should be offered to patients with aggressive PV until their disease is better controlled to minimise the risk of vascular complications.

### Consent

Informed consent was obtained the participant of this case study.

## Abbreviations

ANA: Antinuclear antibodies
ANCA: Antineutrophil cytoplasmic antibody
PE: Pulmonary embolism
PV: Pemphigus vulgaris
VTE: Venous thromboembolism
